# Assumptions and methods in the Lives Saved Tool (LiST)

**DOI:** 10.1186/1471-2458-11-S3-I1

**Published:** 2011-04-13

**Authors:** Monica J Fox, Reynaldo Martorell, Nynke van den Broek, Neff Walker

**Affiliations:** 1 Department of International Health, Bloomberg School of Public Health, Johns Hopkins University, 615 N Wolfe Street, Baltimore, MD 21205, USA; 2 Hubert Department of Global Health, Rollins School of Public Health, Emory University, 1518 Clifton Road. Atlanta, Georgia 30322, USA; 3 Maternal Newborn Health Unit, Liverpool School of Tropical Medicine, Pembroke Place Liverpool L3 5QA, UK

## 

The Lives Saved Tool (LiST) is a computer-based model that estimates the impact of increasing coverage of interventions on maternal, neonatal and child mortality. The model has its origins in earlier work from the Lancet Series papers that looked at estimating the impact of increasing coverage of proven interventions on child mortality [1] and neonatal mortality [2] as well as the impact of interventions related to nutrition and nutritional status of mothers and children [3]. During the past four years, LiST has been developed into a free, publically available software tool that has been used by programs or organizations to estimate the impact of scaling up different interventions and thereby help in the health planning process. [4-6]

The development of LiST is closely linked to the work of the Child Health Epidemiology Reference Group (CHERG) of WHO and UNICEF. The CHERG provides technical inputs to the assumptions and procedures used in the model and also guides the on-going development of the model. As part of this process, this journal issue, along with a previous supplement [7], is being published to ensure that the methods and assumptions in the model are peer reviewed and made publicly available for comments, criticism and feedback.

In addition to the fact that the model now includes 75 interventions, LiST also continues to expand in terms of the scope of the program, including two major functionality additions in the current version. First, the new version of LiST estimates the impact of interventions on stillbirths. Second, the new version of LiST allows users the ability to add future interventions, thereby judging the impact of these interventions in conjunction with existing interventions. For example, one could put in a vaccine for malaria, set the efficacy of the vaccine and then estimate the impact that this vaccine would have on malaria deaths with or without the scale up of an existing malaria intervention, such as insecticide-treated nets or indoor residual spraying. More details about the Lives Saved Tool, (LiST) including documentation, training materials, and background information is available at http://www.jhsph.edu/IIP.

This supplement includes 35 articles, the majority of which present reviews and meta-analyses that are used to estimate the effectiveness of interventions. In addition there are several articles that either look at the possible impact of future interventions or deal with methodological issues related to the Lives Saved Tool.

The effectiveness review articles in the supplement are organized into three broad categories. First, there are seven papers that focus on the impact of interventions on the risk of stillbirth for pregnant women, an output new to LiST. The second section has nine articles that look at the impact of interventions related to maternal, neonatal and child mortality. These articles expand the number of interventions for which effectiveness estimates are available from the previous supplement and also, in the case of rotavirus vaccine, provide an updated estimate of effectiveness based on new trial data.

The third section of the supplement contains nine reviews of nutritional interventions. Previous versions of LiST have used estimates of effectiveness drawn from the Lancet Maternal and Child Undernutrition Series [3], but the papers presented here provide new, updated reviews of the effectiveness of nutrition-related interventions.

The six papers contained in the fourth section of the issue estimate the potential impact of emerging interventions against pneumonia and meningitis utilizing a modified CHRNI methodology [8]. In the past, LiST had a large, but defined set of interventions in the model. Because the interventions discussed in this section are not proven interventions, they are not included in LiST. The most recent version of LiST, however, allows users to enter new or future interventions into the LiST model and then see what additional impact these new interventions would have in conjunction with existing interventions.

The final section of the supplement presents 4 articles that look at methodological issues related to LiST. These papers either compare LiST to other models or measured outputs or provide more detailed explanation of the underlying calculations and methods used in LiST.

The review papers in this supplement provide estimates of the effectiveness for 100 intervention-outcome combinations that were 1) interventions that are already in LiST, 2) new interventions that have been proposed for inclusion in LiST, or 3) pairings of existing interventions to new outcomes, such as stillbirth rate that were not included in earlier versions of LiST.

The quality of the data available to make these estimates varies dramatically. For 23 of the 100 intervention-outcome combinations, the data available to estimate effectiveness were rated as high using a modified GRADE approach.[9] At the other extreme, the estimates of effectiveness for 28 intervention-outcome combinations were made by using a Delphi approach [10] because there were insufficient data available from existing studies. This is in effect a method to seek consensus expert opinion. For the remaining 49 intervention-outcome combinations, the reviewers considered the estimates of effect to be based on studies with low or moderate levels of data quality.

Based on these reviews, some intervention-outcome combinations, such as those where there was a non-significant effect, will not be entered into LiST. Likewise, while Delphi analyses were run for all combinations when insufficient data were available to do a meta-analysis, many of these intervention–outcome combinations will not be included in LiST. These estimates will only be used in cases where there is overwhelming belief in the efficacy of an intervention and ethical constraints rule out studies to estimate the effects. For example, it is difficult to directly estimate the impact of caesarean-sections on cause-specific maternal and neonatal mortality or the effect of skilled birth attendance on maternal and newborn outcomes and it is unlikely that controlled trials can be designed to investigate these issues. For these situations, LiST will use the estimates of effectiveness from the Delphi processes and/or use historical data.

Similarly, in some cases such as the availability and uptake of Emergency (or Essential) Obstetric Care, the intervention consists of a ‘package’ of multiple possible separate interventions. Evaluation of the effect of such complex packages is difficult and should not be confused with the effect (or not) of the individual components of the package on the sought outcomes. Finally it must be recognized that in many studies the sought outcomes are not well defined e.g. a number of studies report on perinatal mortality (using a variety of definitions) but do not separately report on stillbirths as outcome.

Thus, the reviews presented here not only serve to improve the scope and the quality of the assumptions of the Lives Saved Tool, but they also highlight areas where further research is needed on effectiveness of interventions. Many of the interventions where insufficient data exist to estimate impact are being used in countries and/or promoted for wider use. Clearly if better health policy decisions are to be made, additional efforts to collect data on the efficacy and effectiveness of these interventions needs to be prioritized.

## Competing interests

The authors declare that they have no competing interests.

## Contributions

The authors all served as editors for this special issue. Nynke van den Broek served as the editor for the papers that estimated effectiveness of interventions on stillbirths. Reynaldo Martorell served as editor for the articles related to nutritional interventions or outcomes. Neff Walker served as editor for the articles on interventions related to maternal, neonatal and child outcomes as well as the methodological articles. Monica Fox served as the editor for the papers related to the impact of new and emerging interventions as well as serving as the overall organizational editor.

## Pre-publication history

The pre-publication history for this paper can be accessed here:

http://www.biomedcentral.com/1471-2458/null/null/prepub
